# Predicting Cognitive Outcome Through Nutrition and Health Markers Using Supervised Machine Learning

**DOI:** 10.1016/j.tjnut.2025.05.003

**Published:** 2025-05-12

**Authors:** Shreya Verma, Tori A Holthaus, Shelby Martell, Hannah D Holscher, Ruoqing Zhu, Naiman A Khan

**Affiliations:** 1 Health and Kinesiology, University of Illinois Urbana-Champaign, Urbana, IL, United States; 2 Division of Nutritional Sciences, University of Illinois Urbana-Champaign, Urbana, IL, United States; 3 Neuroscience Program, University of Illinois Urbana-Champaign, Urbana, IL, United States; 4 Department of Food Science and Human Nutrition, University of Illinois Urbana-Champaign, Urbana, IL, United States; 5 Department of Statistics, University of Illinois Urbana-Champaign, Urbana, IL, United States; 6 Personalized Nutrition Initiative, University of Illinois Urbana-Champaign, Urbana, IL, United States; 7 Beckman Institute of Advanced Science and Technology, University of Illinois Urbana-Champaign, Urbana, IL, United States

**Keywords:** cognitive function, dietary patterns, MIND diet, personalized health, random forest

## Abstract

**Background:**

Machine learning (ML) use in health research is growing, yet its application to predict cognitive outcomes using diverse health indicators is underinvestigated.

**Objectives:**

We used ML models to predict cognitive performance based on a set of health and behavioral factors, aiming to identify key contributors to cognitive function for insights into potential personalized interventions.

**Methods:**

Data from 374 adults aged 19–82 y (227 females) were used to develop ML models predicting cognitive performance (reaction time in milliseconds) on a modified Eriksen flanker task. Features included demographics, anthropometric measures, dietary indices (Healthy Eating Index, Dietary Approaches to Stop Hypertension, Mediterranean, and Mediterranean–Dietary Approaches to Stop Hypertension Intervention for Neurodegenerative Delay), self-reported physical activity, and systolic and diastolic blood pressures. The data set was split (80:20) for training and testing. Predictive models (decision trees, random forest, AdaBoost, XGBoost, gradient boosting, linear, ridge, and lasso regression) were used with hyperparameter tuning and crossvalidation. Feature importance was calculated using permutation importance, whereas performance using mean absolute error (MAE) and mean squared error.

**Results:**

Random forest regressor exhibited the best performance, with the lowest MAE (training: 0.66 ms; testing: 0.78 ms) and mean squared error (training: 0.70 ms^2^; testing: 1.05 ms^2^). Age was the most significant feature (score: 0.208), followed by diastolic blood pressure (0.169), BMI (0.079), systolic blood pressure (0.069), and Healthy Eating Index (0.048). Ethnicity (0.005) and sex (0.003) had minimal predictive effect.

**Conclusions:**

Age, blood pressure, and BMI show strong associations with cognitive performance, whereas diet quality has a subtler effect. These findings highlight the potential of ML models for developing personalized interventions and preventive strategies for cognitive decline.

## Introduction

Cognitive health is a vital aspect of overall well-being, influencing an individual’s quality of life, independence, and productivity [1]. As the global population ages, the prevalence of cognitive decline and related disorders is expected to rise significantly [2]. By 2050, the number of people living with dementia worldwide is projected to reach 152 million, nearly triple the current figure of 55 million [3,4]. This alarming trend underscores the urgent need for effective strategies to predict, prevent, and manage cognitive decline.

The intricate relationship between lifestyle factors, health markers, and cognitive function has been an important subject of research in recent years [[5], [6], [7]]. Numerous studies have established links between various health indicators and cognitive performance, suggesting that a holistic approach to health management could potentially mitigate risk of cognitive decline [[8], [9], [10]]. Dietary patterns, particularly those rich in antioxidants, ω-3 fatty acids, and vitamins, have been associated with superior cognitive measures [[11], [12], [13]]. The Mediterranean, Dietary Approaches to Stop Hypertension (DASH), and the Mediterranean–Dietary Approaches to Stop Hypertension Intervention for Neurodegenerative Delay (MIND) diets have been linked to protective effects against cognitive decline and dementia [11,[14], [15], [16], [17], [18], [19]]. The Mediterranean diet, rich in fruits, vegetables, whole grains, olive oil, and fish, has protective effects against cognitive decline [20], whereas the DASH diet, focused on fruits, vegetables, whole grains, and low-fat dairy, aims to lower blood pressure [21,22]. The MIND diet, combining elements of both the Mediterranean and DASH, emphasizes neuroprotective foods like leafy greens, berries, and nuts and has shown promise in reducing risk of Alzheimer disease in older adults [17,23]. Similarly, adherence to the Healthy Eating Index (HEI) has been associated with improvements in cognitive function, especially in older adults [24]. Physical activity, especially aerobic exercise, has been demonstrated to improve cognitive performance and reduce risk of cognitive impairment [[25], [26], [27], [28], [29]]. Cardiovascular health markers and BMI are also related to cognitive function [[30], [31], [32]]. Hypertension and obesity in midlife are associated with an increased risk of cognitive decline and dementia in later life [[33], [34], [35], [36]]. Additionally, socioeconomic factors, including education level and income, have been shown to be related to cognitive outcomes, potentially through their impact on lifestyle choices and access to health care [[37], [38], [39]].

Despite these findings, the complex interplay of these factors and their relative importance in predicting cognitive outcomes remains incompletely understood. Traditional research methodologies have provided valuable insights but often fail to capture the multifaceted nature of this relationship. The emergence of machine learning (ML) techniques offers a promising avenue for addressing this complexity by analyzing large data sets with multiple variables and identifying patterns that may not be apparent through conventional statistical approaches [40,41].

Previous studies have demonstrated the potential of ML in predicting cognitive impairment and its progression to conditions such as Alzheimer disease [[42], [43], [44], [45]]. However, there remains a need for robust models that can provide more accurate and actionable predictions. This study aimed to address this gap by using supervised ML models to predict cognitive performance based on a wide range of health and behavioral factors. Specifically, we focused on predicting reaction time (RT) in a modified Eriksen flanker task [46], a well-established measure of cognitive function that assesses attention and inhibitory control. Our a priori hypothesis was that an ML-based approach, which simultaneously considers multiple health and lifestyle factors, would reveal complex relationships and key predictors of cognitive performance, which may be challenging to identify using conventional statistical methods focused on individual or small groups of factors.

To achieve this, we applied advanced multivariate ML models on an aggregated data set of adults, incorporating features such as demographic information, anthropometric measurements, adherence to various dietary indices, physical activity levels, and blood pressure measurements. Using advanced analytical techniques, we aimed to develop a robust predictive model that can account for the complex interactions between these variables and provide insights into their relative importance in determining cognitive outcomes.

This research aimed to fill the gap in knowledge by providing a deeper understanding of the factors that influence cognitive function, potentially revealing previously unrecognized relationships or confirming the importance of known factors. The development of an accurate predictive model could enable early identification of individuals at risk of cognitive decline, allowing for timely interventions and targeted strategies for maintaining and improving cognitive health.

## Methods

### Study design and participants

Secondary analyses were conducted on baseline data from previous studies involving 476 adults aged 19–82 y. Participants were recruited between 2015 and 2023 from the East-Central region of Illinois using email newsletters, social media postings, radio and television advertisements, and flyers. All study procedures were administered in accordance with the tenets of the Declaration of Helsinki, and protocols were approved by the University of Illinois Urbana-Champaign institutional review board. Table 1 provides a summary of the study details. All individuals provided written informed consent before data collection. Exclusion criteria included pregnancy; lactation; a history of chronic gastrointestinal, metabolic, or neurologic disease; medications to alter bowel function or metabolism; neurologic medications; tobacco use; recent bariatric surgery; and food allergies or intolerances. Health history and demographic information, including approximate annual income, education, race, and ethnicity, were obtained via a survey instrument. Participants with missing RT data (*n* = 91) were excluded from the analyses, yielding a final sample of 385.

**TABLE 1:** Overview of the studies in adult participants aggregated for secondary analyses.

IRB number	Study objective	*n*	Age range (y)
20404	Assessment of relationships between different approaches to studying carotenoid status and factors like age, sex, and BMI that may explain variability in carotenoid levels	50	≥18
16840	Investigate the impact of probiotics and prebiotics on behavioral and biological markers of cognition and stress	46	18–64
21730	Examine the independent and combined effects of prebiotic fiber supplementation and exercise on the gut microbiome and human health	31	20–45
16071	Investigate the relationships between cognitive function, metabolic syndrome risk factors, and gut intestinal microbial composition	43	18–44
16277	Investigating the effects of avocados on abdominal obesity, glycemic control, gut microbiota composition, and cognitive function in adults with overweight and obesity	189	18–64
21839	Understand the potential benefits of consuming dietary fiber for broader health outcomes including both gastrointestinal and cognitive health	75	45–75
23219	Examine the relationship between vascular health, stress, and executive functions and memory in adults	42	18–75

### Dietary assessment and diet indices

Dietary intake data were collected and analyzed using the Diet History Questionnaire, versions II and III (2010 and 2018, respectively), developed by the National Cancer Institute [[47], [48], [49]]. To assess adherence to dietary patterns, we used indices frequently used in observational studies: the MIND, Mediterranean, DASH, and HEI-2020 diets. The MIND diet index is scored from 0 to 15, the Mediterranean diet index from 0 to 55, the DASH diet index from 8 to 40, and the HEI-2020 from 0 to 100. The protocol for extracting and scoring diet indices followed methods as previously described [17,22,[50], [51], [52]]. Dietary intakes reported in the Diet History Questionnaire were converted to weekly servings using standard conversion factors and analyzed using Diet∗Calc Analysis Software, version 1.5.0 (January 2015) [53,54]. This comprehensive dietary assessment enabled the evaluation of participants’ adherence to these dietary patterns and energy intake, which were then used in modeling cognitive performance outcomes.

### Physical activity

Physical activity was assessed using the Godin Leisure-Time Exercise Questionnaire (GLTEQ), a validated self-report instrument designed to measure the frequency and intensity of exercise during free time [55]. Participants reported the number of times per week they engaged in strenuous, moderate, and mild exercise for >15 min. The frequency of each type of activity was multiplied by its corresponding metabolic equivalent value—9 for strenuous, 5 for moderate, and 3 for mild activities—to calculate a total leisure activity score. This score provided an estimate of the participants’ overall physical activity levels, which were then used as a feature in the modeling.

### Anthropometrics and blood pressure

Anthropometric measurements, including weight, height, and waist circumference (WC), were obtained in triplicate using standardized procedures. Participants’ height and weight were measured, while wearing lightweight clothing and without shoes using a stadiometer (model 240; SECA) and a digital scale (Tanita WB-300 Plus). BMI was calculated as weight divided by height (kg/m^2^), using the average of the 3 height and weight measurements. WC was measured at the midpoint between the lower margin of the last palpable rib and the top of the iliac crest using a flexible, nonstretchable tape measure [56]. Seated systolic blood pressure (SBP) and diastolic blood pressure (DBP) were measured using automated sphygmomanometers (Omron).

### Cognitive performance assessment

Cognitive performance was assessed using the modified Eriksen flanker task, a widely recognized measure of cognitive function that evaluates attention and inhibitory control [46,57,58]. Participants were presented with a series of trials in which a central target arrow was flanked by 4 distractor arrows pointing in either the same direction (congruent trials) or the opposite direction (incongruent trials). They were instructed to respond to the direction of the central target arrow as quickly and accurately as possible, whereas ignoring the adjacent flanking distractors. Responses were recorded on a 4-button response pad (Current Designs). The task began with 40 practice trials to familiarize participants with the procedure, followed by 2 blocks of 100 randomly assigned experimental trials with equal probabilities. Each stimulus was presented for 83 ms, with a 1000-ms response window. Intertrial intervals were jittered at 1100, 1300, and 1500 ms to minimize anticipatory responses [46]. Behavioral measures of interest included RT for correct responses during both congruent and incongruent trials.

RT was chosen as the outcome measure for this study due to its sensitivity in detecting subtle changes in cognitive processing speed and executive function [57,59,60]. Faster RTs generally reflect better cognitive function, whereas slower RTs can indicate cognitive decline or impairment [61,62]. The average RT for correct responses across both trial types was calculated for each participant, providing a robust measure of cognitive performance.

### Statistical analysis and validation

#### Data preprocessing

Initially, participants with ≥50% missing data (*n* = 11) were excluded from the original sample (*n* = 385) to maintain reliability. Then, the aggregated data set with 374 participants was divided into predictor input features and the predicted outcome. Predictor features included age, sex, ethnicity, income, education level, weight, height, WC, BMI, dietary indices (HEI, DASH, Mediterranean, and MIND diets), SBP, DBP, and physical activity assessed using the GLTEQ. The target outcome was the RT on the modified Eriksen flanker task. With 18 predictors, the resulting sample-to-feature ratio (∼20:1) met established thresholds for ML validation studies in clinical prediction modeling [63].

Within the final data set (*n* = 374), data from participants with ≤20% missing values (*n* = 24) were imputed using the k-nearest neighbor imputation method due to its ability to preserve the multivariate structure of the data set and robustness to different patterns of missing values [64]. Following imputation, categorical data were encoded to convert them into a format suitable for ML algorithms. Nominal categorical data, such as sex and ethnicity, were 1-hot encoded to transform them into a binary matrix, ensuring no ordinal relationships were implied. Ordinal categorical data, such as education and income levels, were label encoded to maintain the ordinal relationships between categories. Continuous data features were standardized by removing the mean and scaling to unit variance using a standard scaler. This step was essential to ensure that all features contributed equally to the analysis and to improve the performance of the ML models by preventing features with larger scales from dominating the learning process [65].

#### Model workflow and analysis

The primary aim of this study was to develop and validate supervised ML models to predict cognitive performance based on various factors. To address this aim, the preprocessed data set (*n* = 374) was split into training (80%) and testing (20%) sets using Scikit-learn [66], with a random shuffle to ensure the generalizability of the models and make them less prone to biases or overfitting. The training set was used to fit the models, whereas the testing set was reserved for evaluating model performance.

Several ML algorithms were used on the training data set, including decision trees, random forest, AdaBoost, XGBoost, gradient boosting, linear, ridge, and lasso regression [67]. These algorithms were selected for their robustness and ability to handle complex interactions between features. Decision trees were selected for their interpretability in allowing clear, rule-based insights, being helpful for understanding how features influence predictions [68]. Random forest was used for its ability to handle large data sets and reduce overfitting by averaging multiple decision trees, improving regression accuracy [69]. AdaBoost and gradient boosting models were used to capture subtle patterns in the data by sequentially building models, where gradient boosting provides finer error minimization [70,71]. XGBoost was selected for its computational efficiency and scalability in gradient boosting, providing a stronger predictive performance [72]. We also tested simpler models, with linear regression serving as a baseline for evaluating the added value of complex models. Lasso regression was used to enhance interpretability and reduce dimensionality by selecting relevant features, whereas ridge regression addressed multicollinearity by shrinking coefficients to improve prediction stability.

Each model was evaluated using k-fold crossvalidation to ensure generalizability and to prevent overfitting. K-fold crossvalidation involves dividing the training data into k subsets, training the model on k − 1 subsets, and validating it on the remaining subset. This process is repeated k times, with the results averaged to provide a robust estimate of model performance, maximizing the use of available data for both training and validation [73]. Hyperparameter tuning was performed using GridSearchCV, which systematically searches for the best hyperparameters by evaluating each combination’s performance through crossvalidation [66,74]. The parameter grid for GridSearchCV was defined for each model, specifying a range of values for key hyperparameters. This approach allowed us to identify the optimal model configuration that maximized performance. Detailed information on the hyperparameters and their ranges can be found in Supplemental Tables 1–6, which provide an overview of the grid search process and results for each model.

Model performance was assessed using mean squared error (MSE) and mean absolute error (MAE). MSE measures the average squared difference between predicted and actual values, with lower MSE indicating better model performance. MAE provides an additional performance metric by measuring the average absolute difference between predicted and actual values.

#### Feature importance

The secondary aim of this study was to determine the relative importance of health and behavioral factors in predicting cognitive performance. Feature importance scores were computed using the permutation importance method on the best-performing model. This method assesses each feature’s significance by shuffling its values and observing the impact on the model’s performance [75], identifying features that significantly increase prediction error when altered [76]. To calculate importance scores, each feature’s values are permuted iteratively for 10 repeats on the test set whereas keeping other variables unchanged. The difference between the unshuffled data and the permuted error represents the feature’s importance, with larger increases indicating stronger predictive power. To ensure reliability, this analysis was restricted to the best-performing model, as suboptimal models risk misrepresenting feature importance due to inherent inaccuracies.

#### Software and tools

The analysis was conducted using Python version 3.11.5, using libraries such as scikit-learn (version 1.3.0) for ML, pandas (version 2.0.3) for data manipulation, seaborn (version 0.12.2) for visualization, and numpy (version 1.24.3) for numerical computations [66,77]. Jupyter Notebook (version 6.5.4) was used for interactive coding and visualization, facilitating reproducibility and transparency in the analysis process.

## Results

### Participant characteristics

**FIGURE 1:**
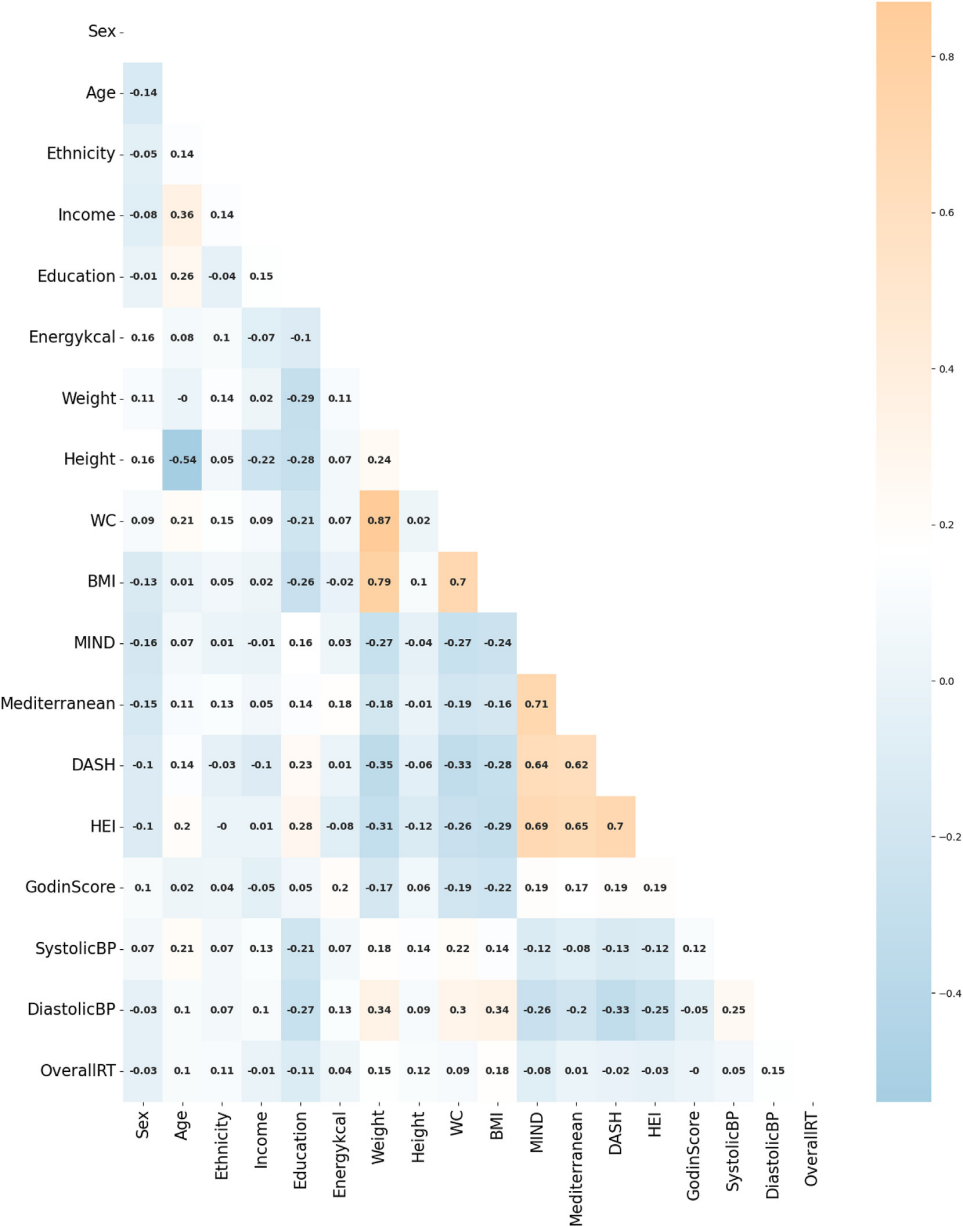
Correlation heatmap illustrating relationships between dietary indices, health markers, and reaction time. This heatmap depicts the correlation coefficients (*r*) between various dietary indices (e.g. MIND, Mediterranean, DASH, and HEI), health-related variables (eg, BMI, WC, and blood pressure), and reaction time (OverallRT) among participants. Blue shades indicate negative correlations, whereas orange shades represent positive correlations, with the color intensity reflecting the strength of the correlation, as shown on the accompanying scale bar. For instance, BMI shows strong positive correlations with WC and weight, indicating expected associations between these physical health markers, whereas dietary indices (MIND, Mediterranean, DASH, and HEI) are positively interrelated, suggesting similar dietary patterns. This analysis aids in understanding the interrelationships among dietary patterns, physical health metrics, and cognitive function markers. BP, blood pressure; DASH, Dietary Approaches to Stop Hypertension; GLTEQ, Godin Leisure-Time Exercise Questionnaire; HEI, Healthy Eating Index; MIND, Mediterranean-DASH Intervention for Neurodegenerative Delay; RT, reaction time; WC, waist circumference.

The data set with 374 participants (227 females, 147 males) aged 19–82 y, with a mean age of 36.98 ± 12.12 y was used for the analyses. The mean RT was 437.28 ± 52.82 ms. Descriptive statistics for the primary features used in the analysis are summarized in Table 2. Additionally, Figure 1 presents a correlation heatmap illustrating the relationships between the predictors and the outcome variable, offering an overview of the associations within the data set.

**TABLE 2:** Participant characteristics^1^.

Characteristic	Values
*N* (female, male)	374 (227, 147)
Age (y)	36.9 ± 12.1
Energy (kcal)	1840 ± 880
Ethnicity, *n* (%)	
White	268 (71.7)
Asian	58 (15.5)
African American	24 (6.4)
American Indian	3 (0.8)
Mixed	21 (5.6)
Income, *n* (%)	
<$41,000	146 (39.0)
$41,000–$80,000	121 (32.4)
>$80,000	107 (28.6)

Weight (kg)	84.2 ± 22.9
Height (cm)	159.6 ± 41.8
Waist circumference (cm)	91.7 ± 15.9
BMI (kg/m^2^)	29.3 ± 7.9
MIND	7.2 ± 2.0
Mediterranean	29.6 ± 6.0
DASH	17.8 ± 4.5
HEI-2020	63.9 ± 11.0
Godin score (GLTEQ)	40.3 ± 36.0
Systolic BP (mm Hg)	114 ±19
Diastolic BP (mm Hg)	81 ± 10
Reaction time (ms)	437.3 ± 52.8

Abbreviations: BP, blood pressure; DASH, Dietary Approaches to Stop Hypertension; GLTEQ, Godin Leisure-Time Exercise Questionnaire; HEI, Healthy Eating Index; MIND, Mediterranean-DASH Intervention for Neurodegenerative Delay; WC, waist circumference.

1 Continuous data presented as mean ± SD where indicated.

### Performance metrics of ML models

The performance of various ML models was evaluated using MSE and MAE on the training and the test data sets; the performance metrics for all models are summarized in Table 3. Random forest emerged as the best-performing predictive model. For the optimization, we conducted hyperparameter tuning using a parameter grid that included variations in the number of estimators, maximum depth, minimum samples split, minimum samples leaf, and bootstrap options. The optimal configuration identified was a random forest regressor with 1000 estimators, a maximum depth of 16, minimum samples split of 5, a minimum samples leaf of 12, and bootstrap enabled [66]. This configuration significantly improved predictive performance, demonstrating its effectiveness in handling the complexity and interactions among the features. The model achieved an MAE of 0.66 ms and an MSE of 0.78 ms^2^ on the training set, and an MAE of 0.70 ms and an MSE of 1.05 ms^2^ on the testing set. The MAE accounts for only 0.16% of the mean RT and 1.3% of its SD, with minimal training–test disparity in MAE (0.04 ms).

**TABLE 3:** Performance metrics of ML models on training and testing data.

Models	Training data		Test data	
	MAE (ms)	MSE (ms^2^)	MAE (ms)	MSE (ms^2^)
Decision tree	0.77	0.98	0.86	1.10
Random forest	0.66	0.78	0.70	1.05
AdaBoost	0.68	0.85	0.78	1.01
Gradient boost	0.67	0.84	0.79	1.02
XG boost	0.67	0.84	0.78	1.02
Linear regression	0.70	0.89	0.84	1.08
Ridge regression	0.73	0.93	0.82	1.07
Lasso regression	0.73	0.93	0.82	1.06

Note: This table presents the performance metrics, including MAE and MSE, for various machine learning models on both the training and testing data sets. Lower values of MAE and MSE indicate better predictive accuracy. The random forest model shows a balanced performance between training and testing data, with the lowest MAE and MSE on the training data and maintaining a relatively low error on the test data.

Abbreviations: MAE, mean absolute error; MSE, mean squared error.

### Feature importance and interaction

The relative importance scores for all features are presented in Table 4 and Supplemental Figure 1. Feature importance scores were computed using the permutation importance method on the optimized random forest model. The results indicated that age (0.208) was the most significant predictor, followed by DBP (0.169), BMI (0.079), SBP (0.069), HEI-2020 score (0.048), and Godin score (0.034). Notably, ethnicity (0.005) and sex (0.003), exhibited minimal predictive power.

**TABLE 4:** Feature importance scores calculated using permutation importance.

Rank	Feature	Importance score
1	Age	0.208
2	Diastolic BP	0.169
3	BMI	0.079
4	Systolic BP	0.069
5	HEI-2020	0.048
6	Godin score (GLTEQ)	0.034
7	Waist circumference	0.027
8	Energy intake (kcal)	0.018
9	Weight	0.017
10	Education	0.017
11	Height	0.016
12	Mediterranean	0.011
13	Income	0.011
14	MIND	0.007
15	DASH	0.007
16	Ethnicity	0.005
17	Sex	0.003

Note: The importance scores represent the relative contribution of each feature to the prediction model. Higher scores indicate greater importance in predicting the target outcome, whereas lower scores suggest a lesser impact. Feature importance was calculated using permutation importance based on the best-performing model, which in this case is the random forest model. Values have been rounded to 3 decimal places to ensure precision in reflecting subtle differences in feature importance across the model.

To further explore the relationship between key predictors and cognitive performance, we conducted 2-way partial dependence analyses for the top-ranked features. Partial dependence plots illustrated the interaction effects of 2 predictors on the predicted outcome while holding all other features constant, revealing conditional patterns that may be obscured in conventional regression models. The analyses revealed interactions between age, blood pressure, BMI, and self-reported physical activity with overall diet quality (Supplemental Figures 2–6). Additional partial dependence analyses demonstrated that higher DASH adherence was associated with faster RTs, particularly in individuals with lower blood pressure (Supplemental Figures 7 and 8). Similarly, greater HEI-2020 adherence partially offset the negative cognitive effects of elevated BMI, highlighting interactive dietary influences on cognition (Supplemental Figure 9).

## Discussion

This study aimed to develop a robust predictive model for cognitive performance using a comprehensive set of health and behavioral factors, with a particular focus on RT in the modified Eriksen flanker task. Analyzing feature importance through the permutation importance method, our results revealed that age, blood pressure, and BMI were the most significant predictors of RT. DBP emerged as more predictive than SBP, and HEI-2020 demonstrated a modest but meaningful contribution to cognitive performance even when stronger physiologic predictors were accounted for, underscoring its relevance as a modifiable dietary factor. These key predictors, identified through supervised ML techniques, may be useful to inform targeted interventions focused on maintaining and enhancing cognitive health.

Our approach involved integrating data from 7 distinct studies that enrolled males and females between the ages of 19 and 82 years, resulting in a diverse and comprehensive data set. We used various ML algorithms, including decision trees, random forest, AdaBoost, XGBoost, gradient boosting, and linear, ridge, and lasso regressions to ensure strong predictive performance. The random forest model achieved the best performance and demonstrated that it fits the training data well and generalizes effectively to unseen data, making it a reliable tool for predicting cognitive performance based on health and behavioral factors. It had the lowest MSE and MAE on the test set and identified age, blood pressure, and BMI as the most influential predictors of RT. Higher age and elevated blood pressure were associated with increased RT, reflecting slower cognitive processing speed. In this context, higher RT is undesirable, as it indicates delayed response time, which can reflect slower mental processing and reduced executive function—factors often linked to impaired quality of life and increased risk of cognitive disorders. Conversely, higher adherence to HEI-2020 was associated with lower RT, suggesting a protective effect of better diet quality on cognitive performance.

The findings reported in this study are consistent with existing literature that emphasizes the influence of age, cardiovascular health, and body composition on cognitive function [[78], [79], [80], [81]]. The results of our modeling approach align with the existing literature indicating that aging is closely associated with cognitive decline [78,79,82]. The importance of blood pressure and BMI also highlights the role of weight status and cardiovascular health in cognitive functioning, consistent with previous findings that link elevated blood pressure and obesity with impaired cognitive performance [36,[82], [83], [84], [85]]. Dietary patterns, particularly adherence to HEI-2020, DASH, MIND, and Mediterranean diets, showed varying levels of influence on cognitive performance, although to a lesser extent than the top predictors. Although the modest importance scores of dietary indices may reflect their secondary predictive role compared with acute physiologic factors, methodological safeguards such as k-fold crossvalidation and permutation importance analysis over multiple iterations ensure these findings are not artifacts of random-effects. The lower rankings likely suggest that dietary influences on cognitive performance are more gradual and cumulative over time, consistent with their classification as modifiable lifestyle factors. These findings position nutrition within a broader integrative framework of cognitive health, emphasizing diet’s interactions with physiologic factors. The importance score of the HEI-2020 highlights the role of overall diet quality in promoting cognitive health. Previous research has demonstrated that adherence to HEI-2020, which reflects alignment with dietary guidelines, is linked to superior executive function and processing speed in older adults [25,86]. Although the Mediterranean and MIND diets also contributed, emphasizing their neuroprotective potential [17,18], their importance was less pronounced than that of the comprehensive HEI-2020. The HEI-2020’s superior predictive performance among dietary indices likely stems from its energy adjustment and emphasis on nutrient-dense foods, which align with neuroprotective mechanisms. Although our study could not examine individual dietary components due to sample size constraints, future studies should explore these granular predictors. Additionally, physical activity was found to be an important predictor of RT, reinforcing the synergistic effects of a healthy lifestyle on cognitive outcomes [26].

The significance of the findings reported in this study lies in their potential to inform targeted strategies for cognitive health management. The low MAE and MSE values observed in our best-performing model indicate strong predictive accuracy, with minimal error relative to the mean RT. These results suggest that the identified predictors provide reliable estimates of cognitive performance. From a translational perspective, these findings reinforce the potential utility of ML models in identifying individuals at higher risk of cognitive decline based on modifiable health factors, which may inform targeted interventions to mitigate risk. Through the identification of key predictors of cognitive performance, personalized interventions aimed at mitigating risk of cognitive decline can be developed. For instance, the interaction between DASH adherence and blood pressure suggests an indirect pathway through which diet may influence cognition, highlighting the potential of interventions targeting blood pressure to help preserve cognitive function in older adults. Traditional statistical methods often fall short in capturing the complex interplay among numerous features. In contrast, ML algorithms excel at analyzing large data sets with multiple variables, uncovering patterns and relationships that conventional approaches might overlook. In addition to identifying key predictors, we also used 2-way partial dependence plots to visualize and understand the interaction effects between pairs of the top predictor variables on the outcome.

The interaction plots demonstrated that higher age and blood pressure are generally associated with slower RTs, particularly when combined with higher BMI. Additionally, the synergy between physical activity and adherence to a healthy diet (as measured by the HEI-2020) highlighted the importance of maintaining both an active lifestyle and a balanced diet for optimal cognitive function. Additionally, higher DASH adherence was associated with better cognitive performance across blood pressure levels, although its benefits were most pronounced at lower blood pressure. Similarly, HEI-2020 adherence helped offset some of the cognitive slowing associated with higher BMI, emphasizing the importance of diet quality even in the presence of other risk factors. These findings suggest that cognitive performance is influenced by a complex interplay of multiple factors, with certain combinations exacerbating or mitigating the effects on cognitive outcomes. The results emphasize the importance of considering both health and behavioral factors in cognitive health management. Although age remains a critical factor beyond control, cardiovascular health emerges as a key area for intervention through diet and physical activity.

The findings from this study have implications for the field of precision nutrition and public health. Although our findings reinforce known associations, this study offers a novel contribution to nutrition science by using ML to quantify the influence of multiple health and dietary factors to capture complex, nonlinear interactions without the constraints of traditional regression modeling. By identifying key factors associated with cognitive function, personalized dietary and lifestyle interventions can be developed to target these factors effectively. For example, personalized strategies focusing on managing blood pressure, maintaining healthy BMI, and promoting physical activity could be prioritized for individuals at a higher risk of cognitive decline. Thus, overall, these findings contribute to advancing a systems-based approach in nutritional science by contextualizing diet within a broader network of cognitive health determinants.

However, this study has several limitations. First, the cross-sectional design limits our ability to infer causality. Longitudinal and experimental studies are needed to confirm these findings and explore the causal and synergistic relationships between the features and cognitive performance. Second, although our predictor variables data set was large, it likely did not capture all relevant variables influencing RT. Future research should consider additional factors such as genetic factors and detailed metabolic profiles. Moreover, the generalizability of our findings may be limited by the demographic characteristics of our sample. Further studies should aim to include more diverse populations to validate the application of our predictive model across different groups. Additionally, although our sample size (*n* = 374) meets established methodological guidelines for ML validation and minimizes overfitting risk, larger data sets may further enhance the stability and generalizability of our findings. Another limitation of our study is the variation in adherence to dietary patterns, with participants showing the highest adherence to HEI-2020 and the lowest to the DASH diet. Future studies should explore barriers to DASH adherence and develop strategies to promote this pattern, enabling a more comprehensive evaluation of its potential cognitive benefits. Finally, this study focused solely on predicting RT; future research should explore the potential of ML models to predict other cognitive outcomes using larger data sets, which could enhance the robustness of the findings.

In conclusion, this work demonstrates the utility of ML models in understanding determinants of cognitive function and underscores the importance of incorporating a range of health indicators in predictive modeling. Through the application of advanced ML techniques, our research contributes to the evolving field of precision nutrition and personalized health care, with the goal of enhancing cognitive health outcomes in aging populations. Future studies should aim not only to validate these results in larger and more diverse cohorts but also to investigate additional factors such as nutrient level contributions to clarify mechanistic pathways and better understand how specific dietary factory influence cognition over time.

## Author contributions

The authors’ responsibilities were as follows – SV: performed analysis, wrote the original draft, and visualized and conceived the study; SV, RZ, NAK: were responsible for funding acquisition. TH, SM, HDH, RZ, NAK, reviewed and edited the manuscript. HDH, RZ, NAK: were responsible for resources. NAK: supervised, investigated, and conceptualized the study; and all authors: read and approved the final manuscript.

## Data availability

Data described in the manuscript, code book, and analytic code will be made available upon request pending application and approval.

## Funding

This research was supported by the Personalized Nutrition Initiative, the National Center for Supercomputing Applications, and the Division of Nutritional Sciences at the University of Illinois Urbana-Champaign, Urbana, IL, United States and the Hass Avocado Board.

## Conflicts of interest

HDH is a member of *The Journal of Nutrition* editorial board and played no role in the Journal’s evaluation of the manuscript. All other authors declare no conflicts of interest.
