# Supplementary material for: Predicting Cognitive Outcome Through Nutrition and Health Markers Using Supervised Machine Learning
Source: J Nutr. 2025 May 12;155(7):2144–53. doi: 10.1016/j.tjnut.2025.05.003 (PMC12308081; doi:10.1016/j.tjnut.2025.05.003)
Supplement: Multimedia component 1 [file mmc1.docx]

**Supplemental Table 1. Parameter tuning- Decision Tree**

| Parameter | Description | Grid Search | Optimal Parameter |
| --- | --- | --- | --- |
| max_depth | maximum depth of the tree | Range: 1-20 | 1 |
| min_samples_split | minimum number of samples required to split an internal node | Range: 2-21 | 2 |
| min_samples_leaf | minimum number of samples required to be at a leaf node | Range 1-11 | 1 |
| min_impurity_decrease | threshold for early stopping in tree growth based on impurity decrease | [0.0, 0.0001, 0.001, 0.01, 0.1] | 0.1 |

**Supplemental Table 2. Parameter tuning- Random Forest with 1000 estimators**

| Parameter | Description | Grid Search | Optimal Parameter |
| --- | --- | --- | --- |
| n_esimators | number of trees in the forest |  | 1000 |
| max_depth | maximum depth of the tree. | 10,12,14,15,16,18, 20, 30, None | 16 |
| min_samples_split | minimum number of samples required to split an internal node | 2,5, 10 | 5 |
| min_samples_leaf | minimum number of samples required to be at a leaf node | 1,2,4,5,6,8,10,12 | 12 |
| Max_features | number of features to consider when looking for the best split | auto, sqrt, log2 | auto |
| bootstrap | whether bootstrap samples are used when building trees | True, False | True |

**Supplemental Table 3. Parameter tuning- Ada Boost**

| Parameter | Description | Grid Search | Optimal Parameter |
| --- | --- | --- | --- |
| n_estimators | maximum number of estimators at which boosting is terminated |  | 50 |
| learning_rate | weight applied to each regressor at each boosting iteration | 0.01, 0.1, 0.5, 1.0 | 0.01 |
| loss | loss function to use when updating the weights after each boosting iteration | 'linear', 'square', 'exponential' | linear |

**Supplemental Table 4. Parameter tuning- Gradient Boost**

| Parameter | Description | Grid Search | Optimal Parameter |
| --- | --- | --- | --- |
| n_estimators | number of boosting stages to perform |  | 100 |
| max_depth | maximum depth of the individual regression estimators. | 3, 5, 7, 9 | 3 |
| learning_rate | Learning rate shrinks the contribution of each tree by this number | 0.01, 0.1, 0.5 | 0.01 |
| min_samples_split | minimum number of samples required to split an internal node | 2, 5, 10 | 10 |
| min_samples_leaf | minimum number of samples required to be at a leaf node | 1,2,4 | 4 |
| subsample | fraction of samples to be used for fitting the individual base learners | 0.8, 0.9, 1.0 | 1 |

**Supplemental Table 5. Parameter tuning- XG Boost**

| Parameter | Description | Grid Search | Optimal Parameter |
| --- | --- | --- | --- |
| n_estimators | number of boosting stages to perform |  | 100 |
| max_depth | maximum depth of the individual regression estimators. | 3, 5, 7, 9 | 3 |
| Learning_rate | step size shrinkage used in update to prevent overfitting | 0.01, 0.1, 0.3 | 0.01 |
| min_child_weight | minimum sum of instance weight needed in a child | 1, 3, 5 | 5 |
| colsample_bytree | subsample ratio of columns when constructing each tree | 0.6, 0.8, 1.0 | 1 |
| subsample | subsample ratio of the training instances | 0.8, 0.9, 1.0 | 0.8 |
| gamma | minimum loss reduction required to make a further partition on a leaf node of the tree. | 0, 0.1, 0.2 | 0.2 |

**Supplemental Table 6. Parameter tuning- Ridge Regression and Lasso Regression**

| Parameter | Description | Grid Search | Optimal Parameter |
| --- | --- | --- | --- |
| Ridge Regression: alpha: | Constant that multiplies the L2 term, controlling regularization strength | np.logspace(-4, 4, 20) | 206.91 |
| Lasso Regression: alpha: | Constant that multiplies the L1 term, controlling regularization strength | np.logspace(-4, 4, 20) | 0.03 |

**Supplemental Figure 1. Bar plot depicting Feature importance derived from the Random Forest model using Permutation Importance**


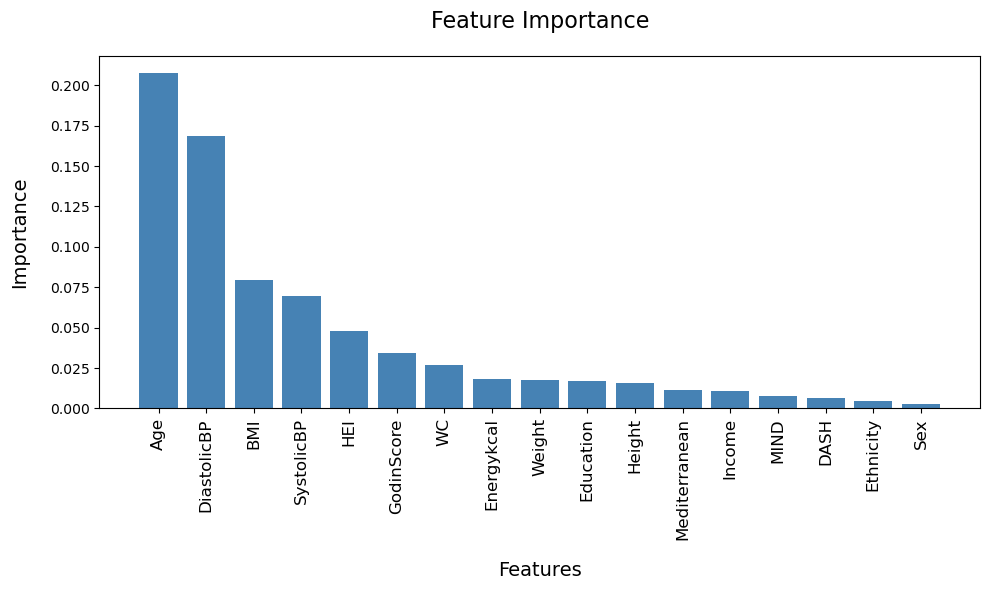


Abbreviations: BP, Blood pressure. DASH, Dietary Approaches to Stop Hypertension. HEI- 2020, Healthy Eating Index 2020. MIND, Mediterranean-DASH Intervention for Neurodegenerative Delay. WC, Waist circumference.

**Supplemental Figure 2. Two-Way Partial Dependence Plot of Diastolic Blood Pressure and Age on Predicted Reaction Time**


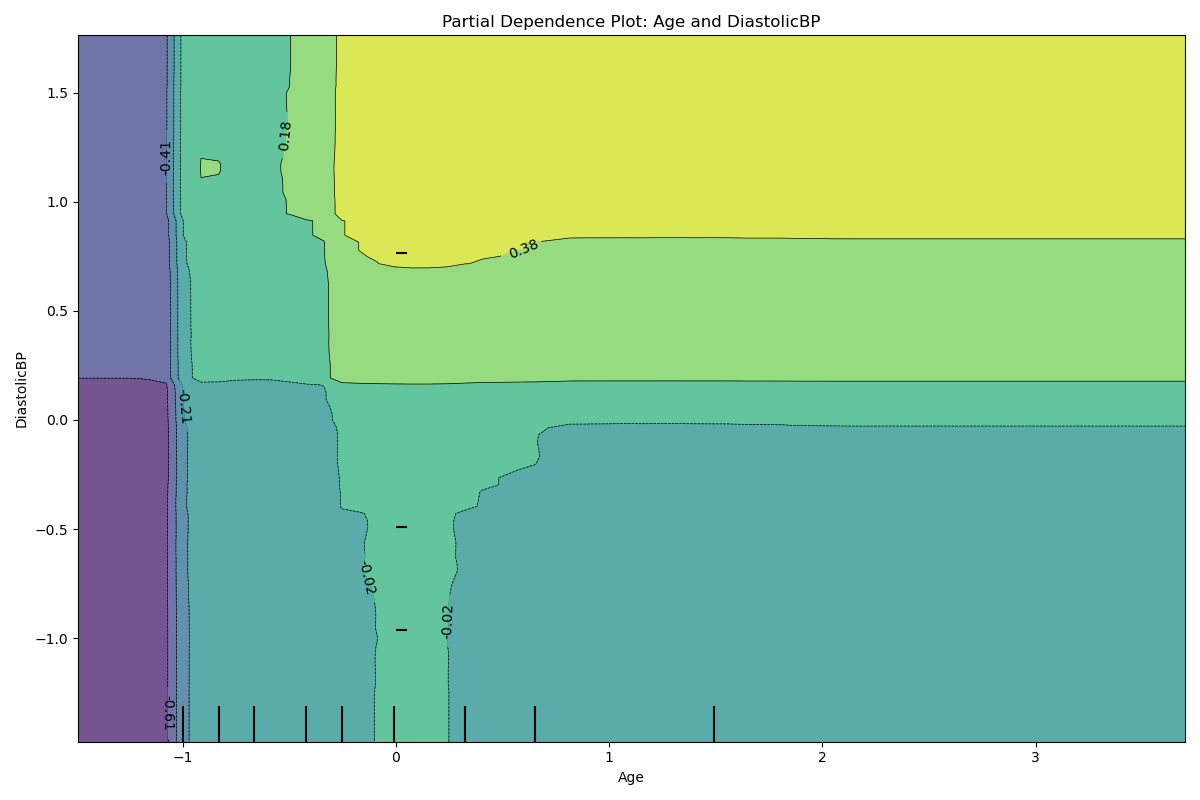


This figure presents a two-way partial dependence plot illustrating the interaction between standardized age and standardized diastolic blood pressure (DBP) in predicting reaction time. The color gradient represents the predicted reaction time, where lighter colors correspond to longer reaction times (slower cognitive processing) and darker colors represent shorter reaction times (faster cognitive processing).

The plot suggests that younger participants with lower DBP (bottom-left corner) generally exhibit shorter reaction times, reflecting better cognitive performance. In contrast, older participants with higher DBP (top-right corner) tend to have longer reaction times, indicating slower cognitive processing. This interaction effect implies that both age and diastolic blood pressure independently and jointly contribute to cognitive outcomes, with aging and elevated blood pressure compounding the risk of cognitive decline. The values for both age and diastolic blood pressure have been standardized, ensuring that the analysis accounts for differences in scale and enhances the interpretability of their combined effects on cognitive performance.

**Supplemental Figure 3. Two-Way Partial Dependence Plot of Systolic Blood Pressure and Age on Predicted Reaction Time**


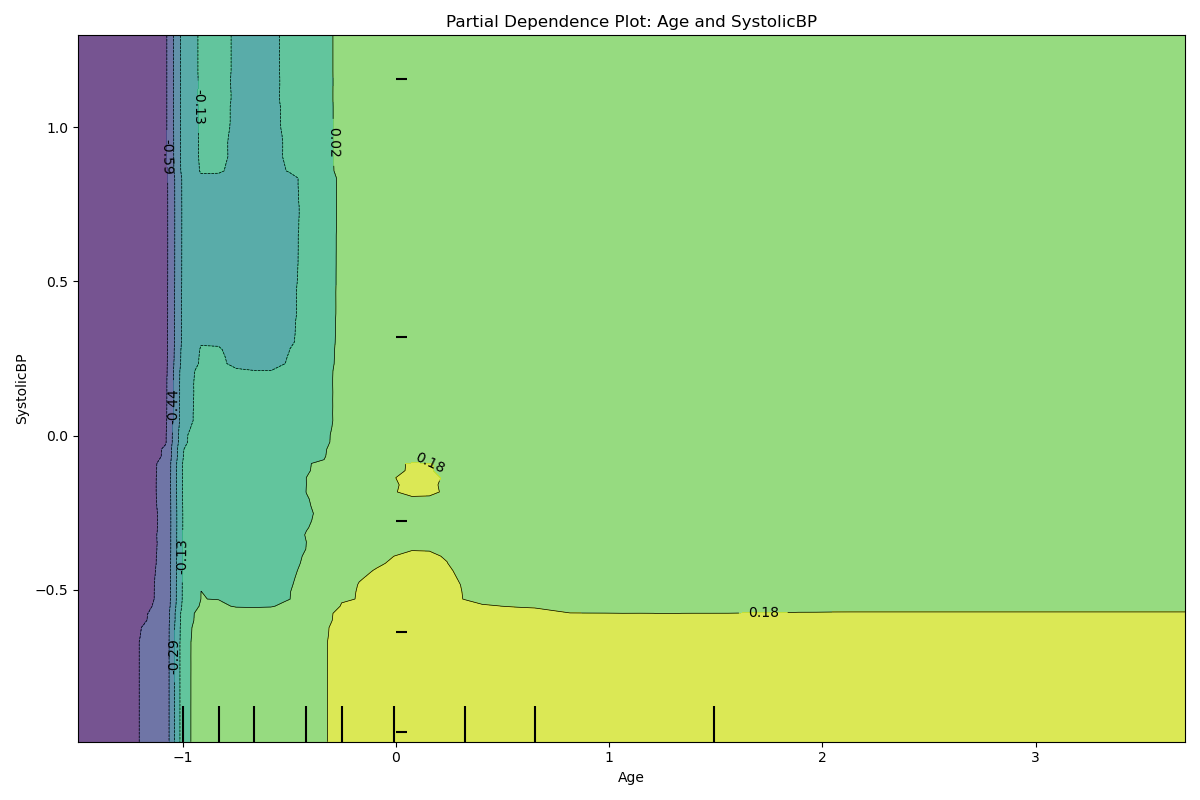


The plot presents a two-way partial dependence plot illustrating the interaction between standardized age and standardized systolic blood pressure (SBP) in predicting reaction time. The color gradient represents the predicted reaction time, with lighter colors indicating longer reaction times (slower cognitive processing) and darker colors denoting shorter reaction times (faster cognitive processing).

The plot shows that younger participants with lower SBP (towards the bottom-left corner) tend to have shorter reaction times, reflecting better cognitive performance. In contrast, older participants with higher SBP (towards the top-right corner) are associated with longer reaction times, indicating slower cognitive processing. This suggests that both age and SBP independently and jointly influence cognitive outcomes.

The interaction effect implies that elevated systolic blood pressure may have a more adverse effect in older individuals, as cognitive performance declines more steeply in this group. Conversely, in younger participants, even moderate increases in SBP do not seem to result in a dramatic decline in cognitive function. These findings highlight the importance of early blood pressure management to preserve cognitive function over the lifespan, as the effect of SBP on cognitive outcomes becomes more pronounced with advancing age. The standardization of both variables ensures that differences in scale are accounted for, enhancing the interpretability of their combined influence on cognitive performance.

**Supplemental Figure 4. Two-Way Partial Dependence Plot of BMI and DBP on Predicted Reaction Time**


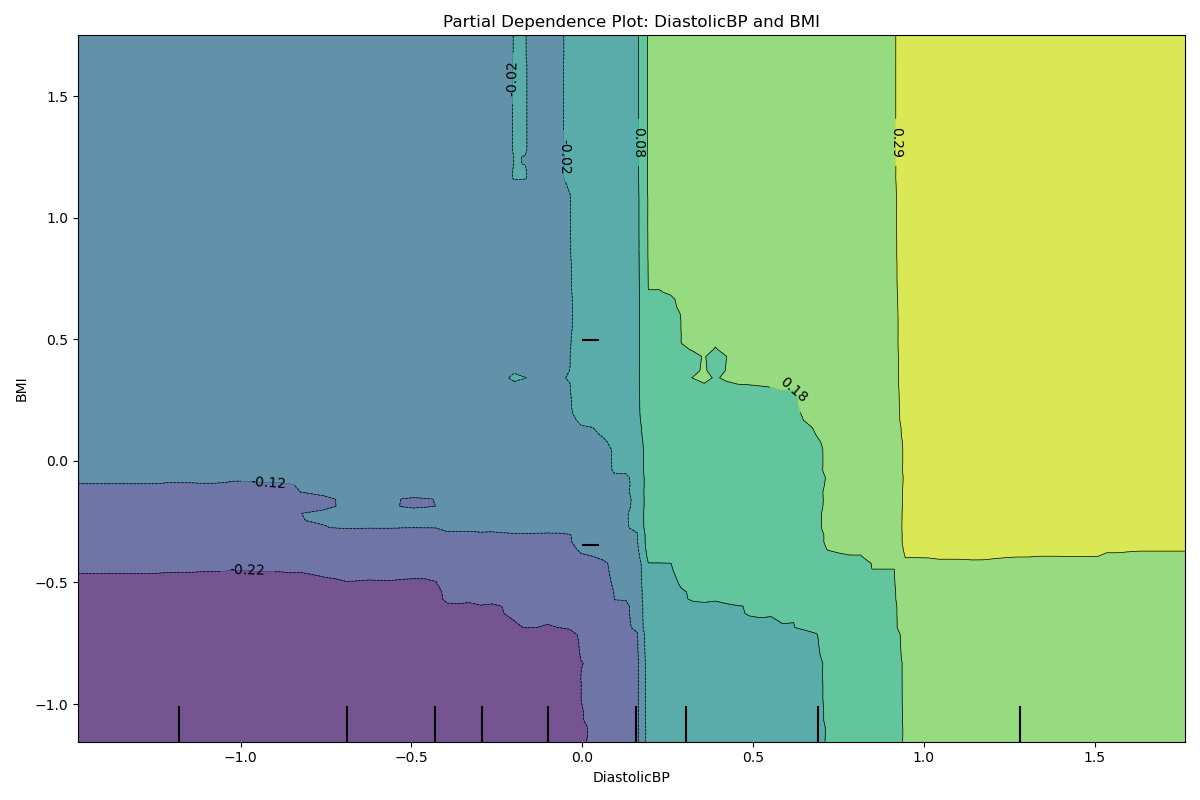


The plot suggests that participants with lower DBP and BMI (towards the bottom-left corner) tend to have shorter reaction times, reflecting better cognitive performance. In contrast, participants with both high DBP and high BMI (towards the top-right corner) are associated with longer reaction times, indicating slower cognitive processing.

This interaction indicates that both DBP and BMI independently and jointly contribute to cognitive outcomes. As DBP and BMI increase, cognitive performance tends to decline, as seen in the lighter areas of the plot. However, participants with low DBP, even with moderate or higher BMI, tend to show better cognitive performance, suggesting that maintaining optimal blood pressure may partially mitigate the cognitive risks associated with higher BMI.

These results highlight the importance of managing both cardiovascular health (DBP) and weight (BMI) to preserve cognitive function. The values for both variables have been standardized to account for differences in scale, ensuring that the combined effects of DBP and BMI on cognitive outcomes are easily interpretable. This underscores the role of integrated health interventions focusing on both blood pressure and weight management to optimize cognitive health.

**Supplemental Figure 5. Two-Way Partial Dependence Plot of Godin (GLTEQ) and HEI on Predicted Reaction Time**


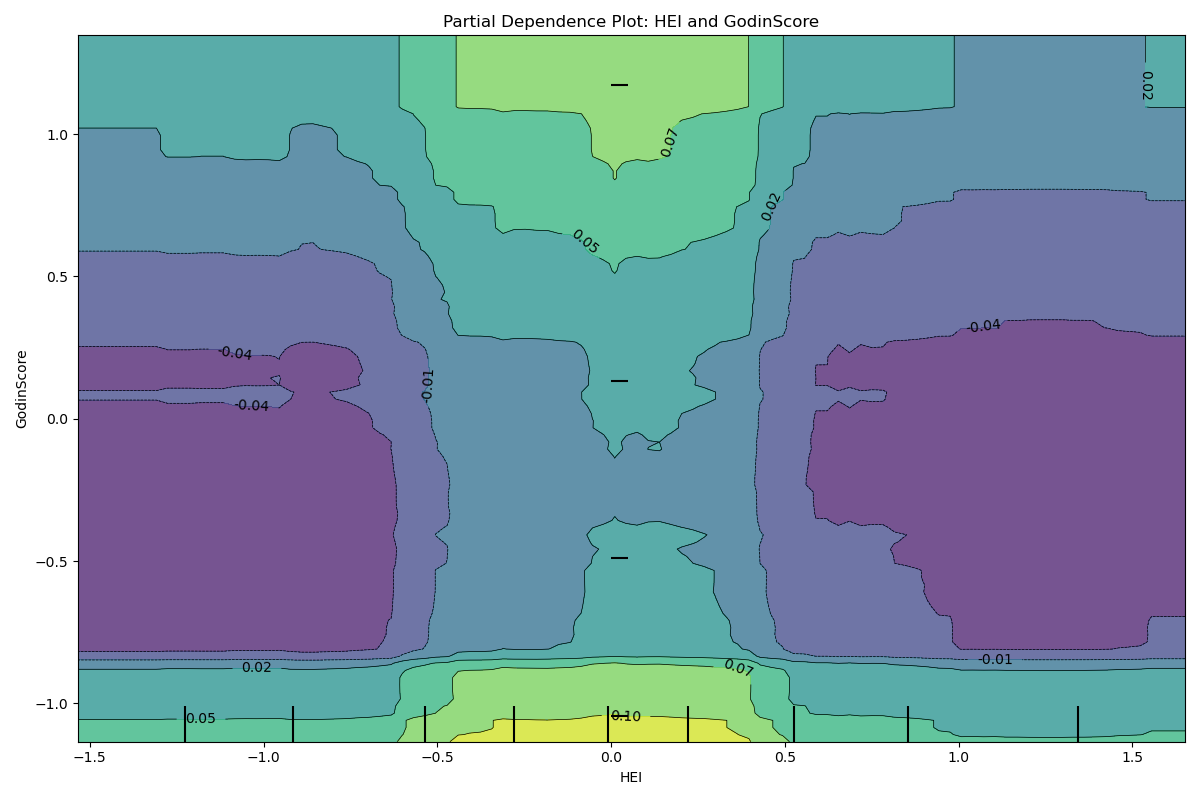


The plot indicates that participants with higher HEI and higher GodinScore (towards the top-right corner) tend to have shorter reaction times, reflecting better cognitive performance. Conversely, low levels of both HEI and GodinScore (bottom-left corner) are associated with longer reaction times, indicating poorer cognitive processing. This suggests that both dietary quality and physical activity independently and jointly influence cognitive outcomes.

Interestingly, individuals with moderate HEI but lower physical activity levels show poorer performance, while those with higher physical activity (even with moderate HEI) exhibit relatively better cognitive performance. This finding highlights the greater impact of physical activity in supporting cognitive function, although the combination of both high physical activity and healthy eating yields the best results.

The interaction effect underscores the importance of maintaining both a healthy diet and an active lifestyle to optimize cognitive performance. The standardization of both variables ensures that differences in their scales are accounted for, allowing for clearer interpretation of their combined effects on cognitive outcomes. These results suggest that interventions focusing on both dietary improvement and increased physical activity may offer the most substantial benefits for cognitive health.

**Supplemental Figure 6. Two-Way Partial Dependence Plot of Age and HEI on Predicted Reaction Time**


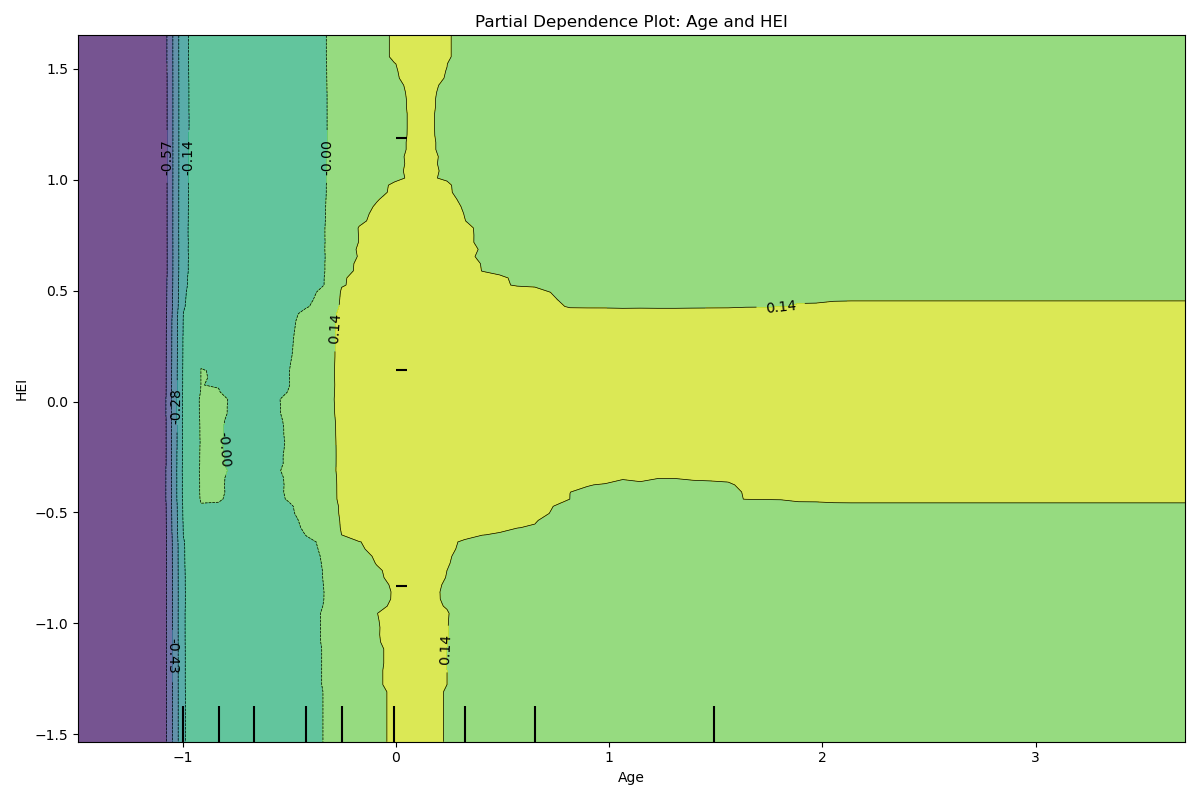


The plot suggests that younger participants with higher HEI adherence (towards the bottom-right corner) tend to exhibit faster reaction times, reflecting better cognitive performance. In contrast, older participants with lower HEI adherence (towards the top-left corner) are associated with longer reaction times, indicating slower cognitive performance.

The plot shows that even participants with higher HEI, but older age do not achieve the same cognitive benefits as younger participants with equivalent dietary adherence. This suggests that diet alone may not fully counteract age-related cognitive decline but still plays an important role in supporting cognitive health.

This interaction indicates that both age and HEI independently and jointly contribute to cognitive outcomes. As age increases, cognitive performance tends to decline, regardless of dietary quality. However, participants who maintain a healthy diet (high HEI) show relatively better cognitive performance across all ages, emphasizing the importance of long-term healthy eating habits for cognitive health.

**Supplemental Figure 7. Two-Way Partial Dependence Plot of Diastolic BP and DASH on Predicted Reaction Time**


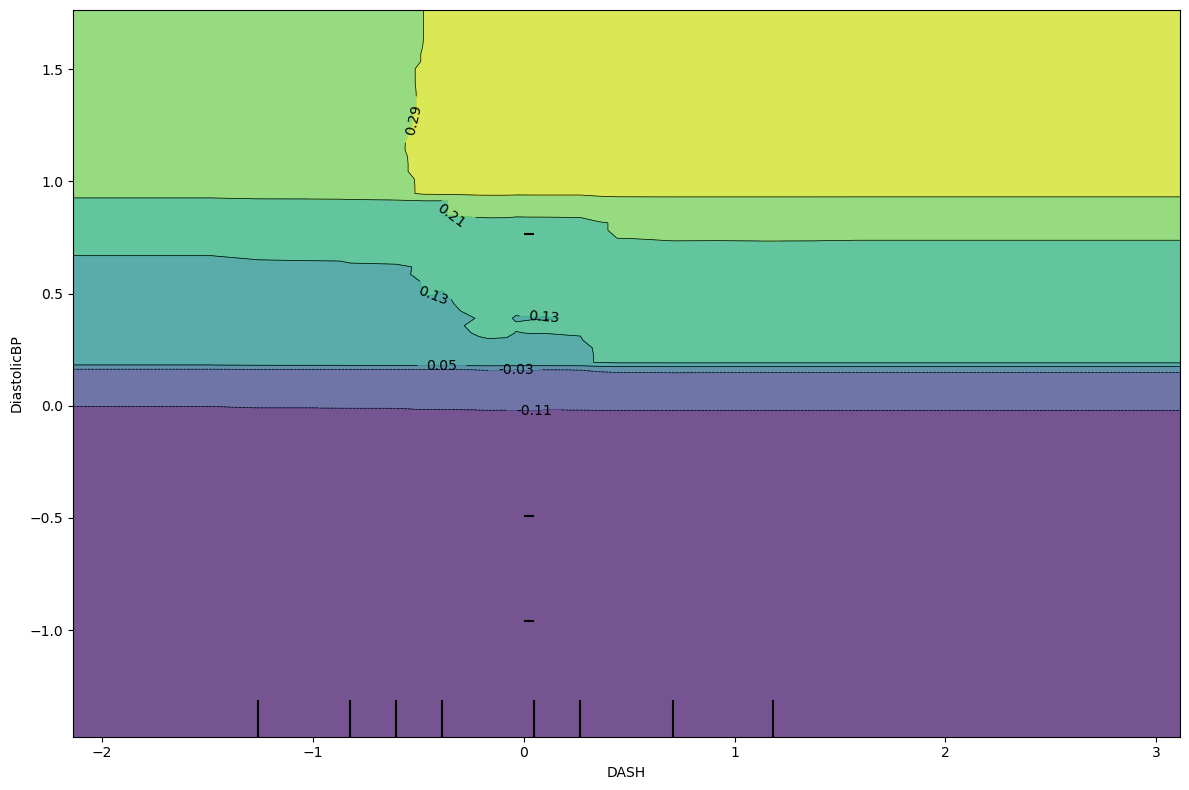


The plot suggests that individuals with lower diastolic blood pressure and higher DASH adherence (bottom-right corner) tend to exhibit faster reaction times, indicating better cognitive performance. In contrast, those with elevated diastolic blood pressure and lower DASH adherence (top-left corner) show slower reaction times, reflecting reduced cognitive efficiency.

The plot shows that even participants with high DASH adherence do not experience the same cognitive benefit if their diastolic blood pressure is substantially elevated. This suggests that while diet quality may help support cognitive health, its positive effects may be limited when vascular health is compromised.

This interaction indicates that both DASH adherence and diastolic blood pressure independently and jointly influence cognitive performance. While higher blood pressure is generally associated with slower cognitive responses, individuals with healthier dietary patterns show relatively better outcomes across all blood pressure levels. This highlights the importance of managing both diet and cardiovascular health to preserve cognitive function.

**Supplemental Figure 8. Two-Way Partial Dependence Plot of Systolic BP and DASH on Predicted Reaction Time**


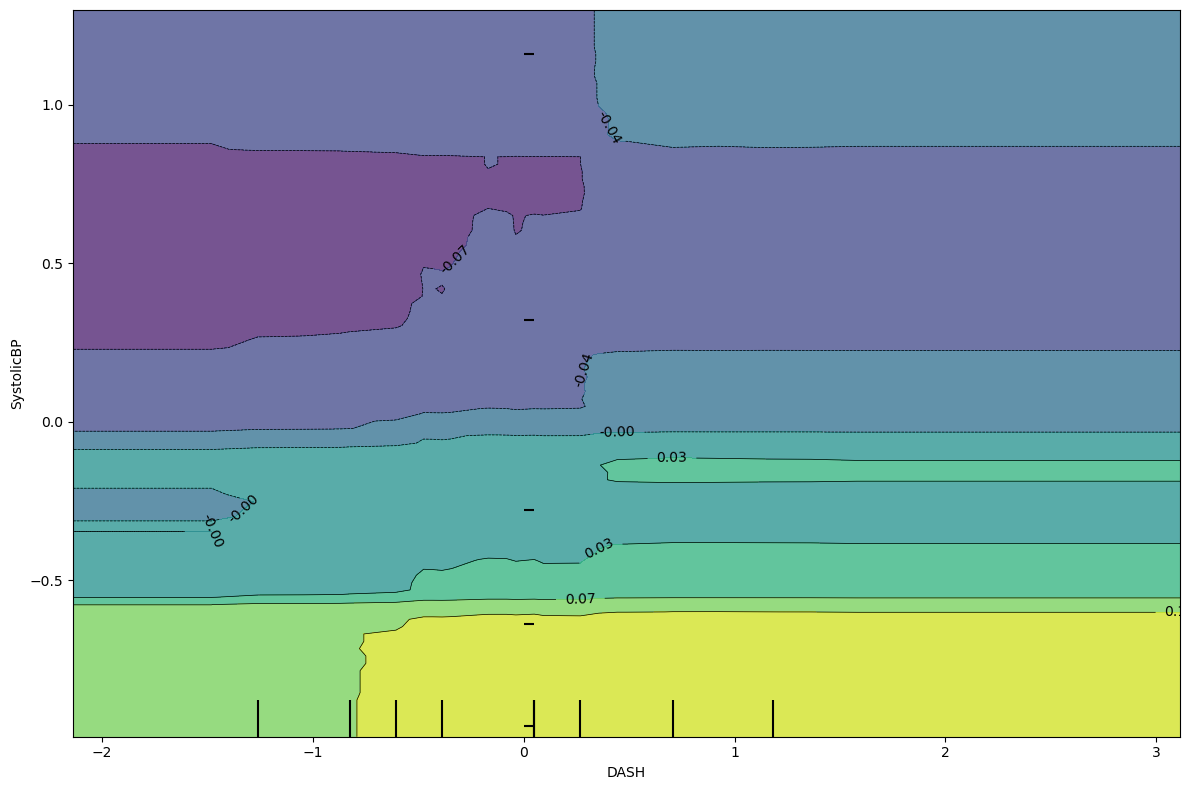


The plot suggests that participants with lower systolic blood pressure and higher DASH adherence (bottom-right corner) tend to exhibit faster reaction times, reflecting better cognitive performance. In contrast, individuals with higher systolic blood pressure and lower DASH adherence (top-left region) show slower reaction times, suggesting reduced cognitive performance.

The plot shows that the positive cognitive effects of DASH adherence are most evident among individuals with lower systolic blood pressure. For those with elevated systolic BP, dietary adherence appears to offer only modest benefits, indicating that the influence of diet may be limited when vascular burden is high.

This interaction suggests that both systolic blood pressure and DASH adherence independently and jointly shape cognitive outcomes. While higher systolic BP is generally associated with slower cognitive responses, participants with healthier dietary patterns tend to perform better across all blood pressure levels. These results reinforce the importance of managing both dietary quality and cardiovascular health to support cognitive performance.

**Supplemental Figure 9. Two-Way Partial Dependence Plot of HEI-2020 and BMI on Predicted Reaction Time**


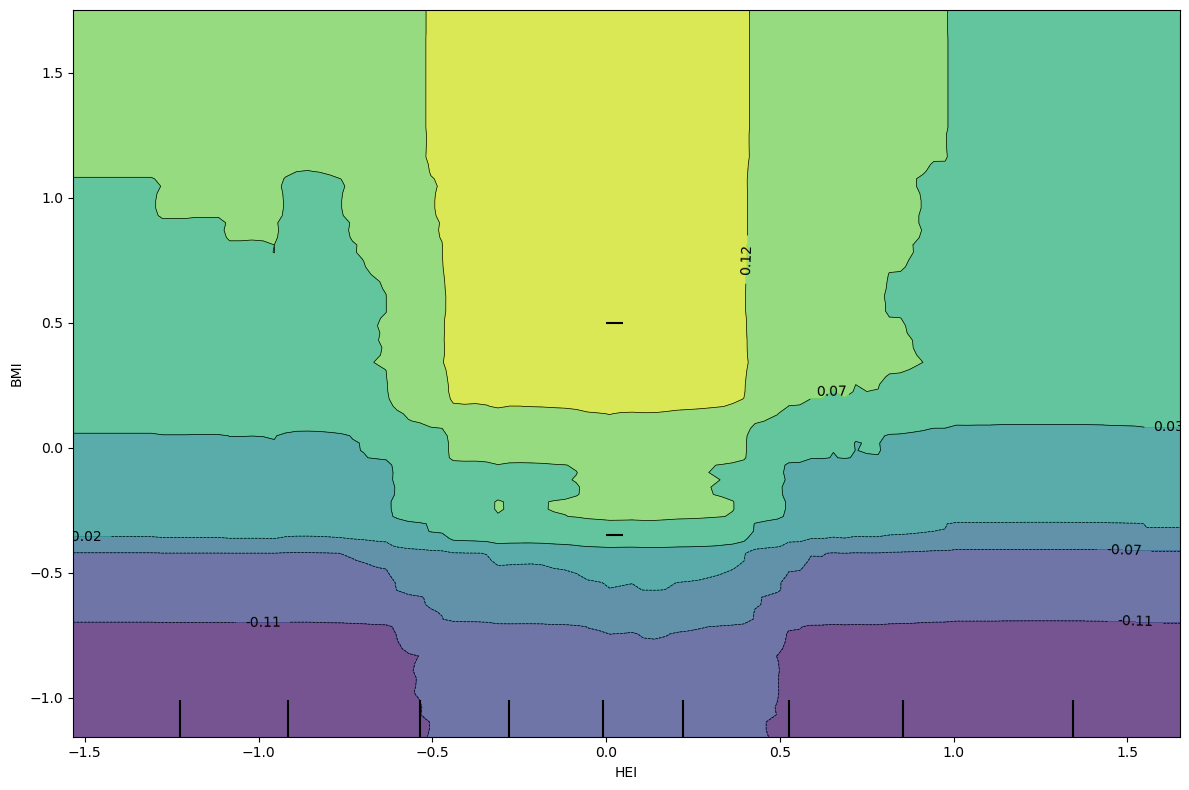


The plot suggests that individuals with lower BMI and higher HEI adherence (bottom-right corner) tend to exhibit faster reaction times, reflecting better cognitive performance. In contrast, participants with moderate-to-high BMI and moderate HEI adherence (center-top region) are associated with slower reaction times, suggesting reduced cognitive efficiency.

It shows that even at higher BMI levels, participants with better HEI scores tend to perform better than those with lower HEI, indicating that healthy eating may help offset some negative effects of elevated BMI. However, the greatest cognitive benefits appear in those with lower BMI, regardless of diet quality.

This interaction suggests that both HEI adherence and BMI independently and jointly shape cognitive performance. While higher BMI is generally associated with slower responses, individuals maintaining a healthier diet show better outcomes across BMI levels. These findings emphasize the importance of both diet quality and weight management in supporting cognitive health.
